# Safety evaluation of allogeneic umbilical cord blood mononuclear cell therapy for degenerative conditions

**DOI:** 10.1186/1479-5876-8-75

**Published:** 2010-08-03

**Authors:** Wan-Zhang Yang, Yun Zhang, Fang Wu, Wei-Ping Min, Boris Minev, Min Zhang, Xiao-Ling Luo, Famela Ramos, Thomas E Ichim, Neil H Riordan, Xiang Hu

**Affiliations:** 1 Nanshan Affiliated Hospital of Guangdong Medical College, Shenzhen, China; 2 Shenzhen Beike Cell Engineering Research Institute, Shenzhen, China; 3 Department of Surgery, University of Western Ontario, London, Ontario, Canada; 4 Department of Medicine, Division of Neurosurgery, University of California San Diego, San Diego, CA, USA; 5 Medistem Inc, San Diego, CA, USA

## Abstract

**Background:**

The current paradigm for cord blood transplantation is that HLA matching and immune suppression are strictly required to prevent graft versus host disease (GVHD). Immunological arguments and historical examples have been made that the use of cord blood for non-hematopoietic activities such as growth factor production, stimulation of angiogenesis, and immune modulation may not require matching or immune suppression.

**Methods:**

114 patients suffering from non-hematopoietic degenerative conditions were treated with non-matched, allogeneic cord blood. Doses of 1-3 × 10^7 ^cord blood mononuclear cells per treatment, with 4-5 treatments both intrathecal and intravenously were performed. Adverse events and hematological, immunological, and biochemical parameters were analyzed for safety evaluation.

**Results:**

No serious adverse effects were reported. Hematological, immunological, and biochemical parameters did not deviate from normal ranges as a result of therapy.

**Conclusion:**

The current hematology-based paradigm of need for matching and immune suppression needs to be revisited when cord blood is used for non-hematopoietic regenerative purposes in immune competent recipients.

## Background

Cord blood mononuclear cells are comprised of a heterogenous population of hematopoietic and mesenchymal stem cells, endothelial progenitor cells, and immature immunological cells [1,2]. The conventional medical use of cord blood is limited to hematopoietic reconstitution [3], with clinical trials ongoing in type I diabetes [4], and cerebral palsy [5]. Preclinical studies have demonstrated efficacy of cord blood in diverse conditions ranging from heat stroke [6,7], to amyotrophic lateral sclerosis [8], to post infarct regeneration [9], to liver failure [10].

In hematopoietic stem cell transplants ablation of recipient marrow is required to eradicate the endogenous stem cell compartment, and HLA matching with post transplant immune suppression is used to prevent GVHD [3]. For non-hematopoietic applications such as cardiovascular or neurological indications, the therapeutic activities of the cord blood are believed to be mediated in many cases by growth factor secretion [11,12], therefore permanent graft survival is not essential. In these situations the use of non-matched, allogeneic cells may be acceptable. The major barrier to this approach is the theoretical fear of inducing GVHD.

From practical experience there is some evidence that in immune competent recipients, non-matched allogeneic cord blood cells do not elicit GVHD. Specifically: a) Recipients of cord blood in the transfusion scenario, in some cases up to 37 units, have not reported GVHD; b) T cells comprise the GVHD-causing component of cord blood. Administration of allogeneic lymphocytes for prevention of recurrent spontaneous abortion has not led to GVHD, despite higher T cell doses than found in cord blood transplants; and c) Despite presence of fetal T cells in mothers, GVHD associated with pregnancy has not been reported [13].

Under the practice of medicine, several treatment facilities have been using cord blood stem cells without matching or immune suppression [14-17]. Despite identification of a "clinical signal", studies have been extremely limited in patient numbers. In the current report we analyzed safety parameters of 114 patients treated with non-matched, allogeneic cord blood mononuclear cells. Treatments included intravenous and intrathecal administration. No immunological reactions, GVHD, or serious adverse effects were observed. Hematological, biochemical, and immunological parameters remained within normal range.

## Methods

### Patient characteristics

Data reported was collected from patients treated during August 2005-July 2007 as part of medical practice at the Nanshan Affiliated Hospital of Guangdong Medical College. All patients were free of: 1) prior history of severe allergic reactions; 2) history of, or active, malignancy; 3) active systemic or severe focal infections (including HIV and syphilis); 4) active cardiac, pulmonary, renal, hepatic or gastrointestinal disease; 5) coagulopathy or any other contraindication for lumbar puncture; 6) gastrostomy, tracheostomy or noninvasive ventilatory support - as these could influence the prognosis and end-point measurements; 7) any severe psychiatric disorder and 8) any immunodeficiency disease or condition.

Age range was 15 to 68 and the male:female ratio was 1.6:1 (70 males, 44 females). In terms of diagnosis, 4 patients had multiple system atrophy (MSA), 23 patients had ataxias, 42 patients were paraplegic, 19 patients had multiple sclerosis, 12 patients had Amyotrophic Lateral Sclerosis (ALS) and 14 patients had other diagnoses (Table 1). The local institutional review board of the Nanshan Affiliated Hospital of Guangdong Medical College, under the auspices of the National Ministry of Heath, approved application of the technique and consent forms were obtained from each patient before initiation of treatment.

**Table 1:** Patients treated by condition

Condition	Number of patients
Paraplegia	42
Ataxia	23
Multiple Sclerosis	19
Amyotrophic Lateral Sclerosis	12
Sequelae of Cerebrovascular Diseases	6
Multiple System Atrophy	4
Motor Neuron Disease	2
Cerebral Palsy	1
Nerve Injury (Brachial plexus)	1
Traumatic Brain Injury Sequelae	1
Hypoxic-ischemic Encephalopathy Sequelae	1
Cervical Spondylotic Myelopathy	1
Optic Nerve Hypoplasia	1

### Cell processing

Umbilical Cord Blood (100~ 150 mL) was collected from healthy unrelated donors (signed an informed consent) in accordance with the sterile procurement guidelines for cord blood in each hospital. After collection, each blood sample was tested for communicable diseases such as HBV, HCV, HIV, ALT, and Syphilis. Cord blood was diluted with saline in the ratio 2:1 and 30 mls of the diluted blood was then added to 15 mls of Ficoll in every 50 ml centrifuge tube and then centrifuged (750 g × 22 minutes). Mononuclear cells were collected and washed twice in saline. Contaminating erythrocytes were lyzed with lysis buffer comprising of injection grade water.

Cell density was adjusted to 2 ~ 6 × 10^6^/ml and seeded in DMEM/F12 culture medium with bFGF and EGF at a concentration of 20 ng/ml. Culture media was mixed with 2% v/v B-27 Stem Cell Culture Supplements. Cells were cultured at 37°C with saturated humidity and 5% CO2 by volume. At this stage, all relevant information about the initial culture is entered in the batch information record including test results for sterility, mycoplasma and endotoxin. Cell growth was regularly monitored and the inspection records updated accordingly. Cells were harvested for clinical application after one week of cultivation with cell quantity ≥1 × 10^7 ^and viability ≥95%.

To ensure the quality of the UCB-derived mononuclear cells, a number of parameters are confirmed before use. These are as follows: 1) Raw material control: Tests (HBV, HCV, HIV, ALT and Syphilis) for communicable diseases for UCB units are carried out before any processing begins. Testing was performed by third party laboratory under local government-monitored conditions.

2) In-process control: Non-qualifying cells were eliminated in accordance with Beike's cell counting and morphology standards which include cell quantity ≥1 × 10^7 ^and the highly homogeneous cells possessing a round shape and non-adherence to the culture flask.

3) Culture control: Any contaminated cell suspensions or unhealthy cells were eliminated upon discovery. Non-contamination was determined as lack of sterility, mycoplasma, and lack of visible microorganisms by microscopy. Furthermore samples had to have an endotoxin level≤0.5 EU/ml and be negative for free DNA.

4) Finished product control: This incorporates a final cell count (≥1 × 10^7^, containing 1.0-2.0% CD34+ cells as determined by flow cytometry), cell viability (≥95%) and sterility test.

### Cell administration

Intrathecal injection by lumbar puncture was combined with intravenous infusion and repeated four or five times - depending on the patient's condition. Treatments were separated by one week intervals. Lumbar puncture was performed in the lateral decubitus position, prepped and draped in sterile fashion, and the needle placed in the lumbar cistern. Two mls of Cerebro-Spinal Fluid (CSF) was removed and replaced by 2 mls of cell suspension containing 1-3 × 10^7 ^cells. A 30 ml intravenous infusion of cell suspension was given through an intravenous catheter in 15-20 minutes.

### Statistics

Adverse events were analyzed for all 114 cases, and are presented as percentage values. For analysis of laboratory parameters, the continuous variables were compared using Student t-test with alpha set at 0.05 by group. When the data set did not conform to the normal distribution, logarithmic transformation was used. Inter-quartile-range (IQR) computation and boxplots were used to detect outliers. The outliers were firmly believed to be data errors or data entry errors and were removed from the data analysis. The SPSS 13.0 statistical package was applied for statistical analysis.

## Results

Administration of cord blood mononuclear cells via intrathecal and intravenous routes was well tolerated. No allergic or immunological reactions were noted at the time of injection or while under observation. Analysis of overall adverse events (Table 2) for a 4-5 week follow-up time period indicated headache as the most common (3.21%). In all cases headaches were transient in nature. No deviation outside of reference ranges was observed for hematological (Table 3), biochemical (Table 4), or immunological (Table 5) measurements. Average follow-up time for post-treatment analysis was 30 days. Some pre and post treatment differences reaching statistical significance were however observed.

**Table 2:** Analysis of adverse events (AE)

AE	Total injections in person time	Number of AE by type (person-time)	Incidence of AE
Headache	592	19	3.21%

Fever	592	7	1.18%

Waist pain	592	5	0.84%

Shivering	592	3	0.51%

Vomiting	592	2	0.34%

Lower limb pain	592	2	0.34%

Total	592	38	6.42

**Table 3:** Hematology

Parameter	Number of patients	Before treatment	After treatment	Reference range	P value	Significance
Leukocytes (×10^9^/L)	114	6.94 (1.57)	7.85 (2.25)	4.0-10.0	<0.001	In normal range but significantly elevated after treatment

Neutrophilic leukocytes % of total leukocytes	114	59.70 (10.39)	65.03 (13.06)	50.0-70.0	0.001	In normal range but significantly elevated after treatment

Lymphocytes % of total leukocytes	114	30.23 (9.20)	26.03 (10.32)	20.0-40.0	<0.001	In normal range but significantly decreased after treatment

RBC (×10^12^/L)	113	4.61 (0.51)	4.47 (0.46)	3.5-5.0	<0.001	In normal range but significantly decreased after treatment

Mean cell hemoglobin (g/L)	113	137.02 (14.54)	132.88 (13.98)	110.0-150.0	<0.001	In normal range but significantly decreased after treatment

Platelets (×10^9^/L)	113	193.94 (47.64)	206.21 (54.52)	100.0-300.0	0.005	In normal range but significantly elevated after treatment

**Table 4:** Serum chemistry

Parameter	Number of patients	Before treatment	After treatment	Reference range	P value	Significance
Total bilirubin (μmol/L)	113	1.13 (0.14)	1.09 (0.15)	0.23-1.35	0.002	In normal range but significantly decreased after treatment

Total protein (g/L)	114	65.03 (5.27)	63.20 (6.27)	60.0-85.0	0.002	In normal range but significantly decreased after treatment

Glutamic-pyruvic transaminase, (GPT) (U/L)	114	1.37 (0.22)	1.33 (0.20)	0.7-1.65	0.037	In normal range but significantly decreased after treatment

Glutamic-oxaloacetic transaminase (GOT) (U/L)	114	23.60 (12.45)	21.01 (8.56)	5.0-45.0	0.005	In normal range but significantly decreased after treatment

Serum urea nitrogen (BUN) (μmol/L)	114	4.63 (1.58)	4.58 (1.88)	2.0-7.1	0.750	In normal range, no significant difference

Serum creatinine (SCR) (μmol/L)	114	1.81 (0.16)	1.81 (0.16)	1.64-2.12	0.898	In normal range, no significant difference

Uric acid (UA) (μmol/L)	114	308.27 (80.88)	309.28 (89.64)	90.0-440.0	0.871	In normal range, no significant difference

**Table 5:** Immunological parameters

Parameter	Number of patients	Before treatment	After treatment	Reference range	P value	Significance
T-cells (CD3)% of total T cells	113	79.91 (6.78)	77.67 (8.18)	61-85	0.001	In normal range but significantly decreased after treatment

Helper T-cell (Th cell/CD4) % of total T cells	114	48.84 (9.03)	45.44 (10.65)	28-58	<0.001	In normal range but significantly decreased after treatment

Ts cell (CD8)% of total T cells	114	25.38 (7.18)	26.89 (8.10)	19-48	0.005	In normal range but significantly increased after treatment

CD4/CD8	114	0.30 (0.20)	0.24 (0.23)	-0.05-0.30	<0.001	In normal range but significantly decreased after treatment

IgG (g/L)	114	0.96 (0.12)	0.91 (0.14)	0.86-1.23	<0.001	In normal range but significantly decreased after treatment

IgA (g/L)	114	2.15 (0.79)	2.01 (0.72)	0.68-3.83	<0.001	In normal range but significantly decreased after treatment

IgM (g/L)	114	1.13 (0.62)	1.32 (0.72)	0.63-2.77	<0.001	In normal range but significantly increased after treatment

Complement C3 (g/L)	114	1.19 (0.23)	1.21 (0.25)	0.85-1.93	0.103	In normal range but no significant changes after treatment

Complement C4 (g/L)	114	-0.62 (0.17)	-0.63 (-0.16)	-0.92 - -0.44	0.283	In normal range but no significant changes after treatment

Slight but statistically significant alterations in mean hematological values were noted. Treatment was associated with increased total leukocyte 6.94 ± 1.57 vs 7.85 ± 2.25, neutrophil 59.70 ± 10.39 vs 65.03 ± 13.06, and platelet 193.94 ± 47.64 vs 206.21 ± 54.52 counts. Reduction in lymphocyte 30.23 ± 9.20 vs 26.03 ± 10.32, RBC4.61 ± 0.51 vs 4.47 ± 0.46, and MCH 137.02 ± 14.54 vs 132.88 ± 13.98 was observed (Table 3).

Total bilirubin 1.13 ± 0.14 vs 1.09 ± 0.15, total protein 65.03 ± 5.27 vs 63.20 ± 6.27, GPT1.37 ± 0.22 vs 1.33 ± 0.20, GOT 23.60 ± 12.45 vs 21.01 ± 8.56, and creatinine 1.81 ± 0.16 vs 1.81 ± 0.16 where significantly decreased after treatment, whereas BUN and uric acid were not altered (Table 4).

CD3 T cells 79.91 ± 6.78 vs 77.67 ± 8.18, CD4 T cells 48.84 ± 9.03 vs 45.44 ± 10.65, and the CD4/CD8 ratio 0.30 ± 0.20 vs 0.24 ± 0.23 were decreased, whereas an increase in CD8 cells was observed with treatment 25.38 ± 7.18 vs 26.89 ± 8.10. Of soluble immune parameters, C3 and C4 were not affected by treatment, whereas IgG 0.96 ± 0.12 vs 0.91 ± 0.14 and IgA 2.15 ± 0.79 vs 2.01 ± 0.72 levels were decreased. An increase in IgM levels 1.13 ± 0.62 vs 1.32 ± 0.72 was noted post treatment (Table 5).

## Discussion

The possibility of using non-matched, allogeneic cord blood cells for regenerative medicine applications in absence of immune suppression would overcome several substantial hurdles existing today in stem cell therapy. Although cord blood derived cells are superior to bone marrow in terms of growth factor production ability, pluripotency, and immune modulating activity [18,19], their use has been limited to autologous sources for regenerative applications. The reason for this is has been the argument that the potential adverse effects of myeloablative therapy outweigh possible regenerative activities. The current study investigated the safety of allogeneic cord blood cells for use in regenerative applications in absence of immune suppression.

No serious adverse effects were observed. The most common adverse reaction reported was headache (3.21%), some of which was believed to be caused by postural hypotensive headaches, which is a known complication of lumbar puncture procedures. These symptoms chronologically followed the treatment, and resolved spontaneously without aggressive intervention. These findings are consistent with a Boston Children's Hospital's study that recorded a similar adverse reaction profile to cryopreserved (CD34+) hematopoietic stem cells in the treatment of children [20]. These incidence rates are also similar to those of the published PBPC and Ficoll groups (grouped by isolation method).

Of the full range of laboratory parameters in the analysis, only the changes of lymphocyte (decreased) and neutrophil (increased) count could be described as medically significant. A key contributing factor to these changes is possibly the fact that most patients received an intravenous injection of dexamethasone (5 mg, once) prior to each stem cell injection, to suppress possible adverse reactions. It has been reported that dexamethasone affects white blood cells, segmented neutrophils and lymphocytes [21], and that dexamethasone at therapeutic doses can have a suppressive effect on the lymphocyte proliferative response.

## Conclusion

In summary, these data support the safety and freedom from immunologically-mediated adverse effects of allogeneic cord blood therapy in absence of immune suppression/myeloablation. This study presents for the first time a detailed safety analysis of using non-matched, allogeneic cord blood cells to treat non-hematopoietic degenerative conditions. The longest follow-up with this protocol was 4 years with no evidence of immune reactivity or GVHD. Evaluation of therapeutic benefit is currently in progress.

## Competing interests

Xiang Hu is a shareholder of Beike Biotechnology. No other authors declare any competing interests.

## Authors' contributions

WY conceived of the study, participated in its design and coordination and carried out the clinical treatment. YZ analyzed and interpreted data and drafted the manuscript. FW carried out the clinical treatment and collected data. WM analyzed data and helped to draft the manuscript. BM participated in the data analysis and helped to draft the manuscript. MZ participated in the design of the study and carried out the clinical treatment. XL carried out the clinical treatment and performed the statistical analysis. TI helped to draft the manuscript. FR, TEI and NR analyzed and interpreted data, performed the statistical analysis and helped to draft the manuscript. XH conceived of the study, participated in its design and coordination and helped to draft the manuscript. All authors read and approved the final manuscript.
